# Uncovering the Quantitative Relationships Among Chromosome Fluctuations, Epigenetics, and Gene Expressions of Transdifferentiation on Waddington Landscape

**DOI:** 10.1002/advs.202103617

**Published:** 2022-02-01

**Authors:** Wen‐Ting Chu, Xiakun Chu, Jin Wang

**Affiliations:** ^1^ State Key Laboratory of Electroanalytical Chemistry Changchun Institute of Applied Chemistry Chinese Academy of Sciences Changchun Jilin 130022 China; ^2^ Department of Chemistry & Physics State University of New York at Stony Brook Stony Brook NY 11794 USA

**Keywords:** chromosome fluctuations, epigenetics, transdifferentiation, Waddington landscape

## Abstract

The 3D spatial organization of the chromosomes appears to be linked to the gene function, which is cell type‐specific. The chromosome structural ensemble switching model (CSESM) is developed by employing a heteropolymer model on different cell types and the important quantitative relationships among the chromosome ensemble, the epigenetic marks, and the gene expressions are uncovered, that both chromosome fluctuation and epigenetic marks have strong linear correlations with the gene expressions. The results support that the two compartments have different behaviors, corresponding to the relatively sparse and fluctuating phase (compartment A) and the relatively dense and stable phase (compartment B). Importantly, through the investigation of the transdifferentiation processes between the peripheral blood mononuclear cell (PBMC) and the bipolar neuron (BN), a quantitative description for the transdifferentiation is provided, which can be linked to the Waddington landscape. In addition, compared to the direct transdifferentiation between PBMC and BN, the transdifferentiation via the intermediate state neural progenitor cell (NPC) follows a different path (an “uphill” followed by a “downhill”). These theoretical studies bridge the gap among the chromosome fluctuations/ensembles, the epigenetics, and gene expressions in determining the cell fate.

## Graphical abstract

The chromosome has different phase behaviors on the two compartments A and B. It can be shown by the chromosome structural ensemble switching model that there is a quantitative relationship among gene expressions, epigenetics, and chromosome ensemble structural fluctuations. In transdifferentiation, the switch of epigenetic marks can have a significant effect on the chromosome structural ensemble and gene expression.

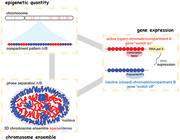

## 1 Introduction

The linkage between the genome and the life is the most significant, fundamental, and complicated issue of biological science. During the recent 20 years, the developments of several essential projects, including the Human Genome Project,^[^ ^1^ ^]^ the encyclopedia of DNA elements (ENCODE),^[^ ^2^ ^]^ and the 4D nucleome project^[^ ^3^ ^]^ have contributed significantly to the understanding of the genomics and cell fate, and paved a promising road for the effective prevention and treatment of human diseases. The techniques of 3C, 4C, and Hi‐C, in particular the single‐cell Hi‐C have shown that the 3D chromosome structures are flexible and vary with the developmental stages, mutants, and cell types.^[^ ^4^, ^5^, ^6^, ^7^, ^8^, ^9^ ^]^ The ensemble‐average behaviors of chromatin can be captured by the Hi‐C contact frequency map. The Hi‐C method combines DNA proximity ligation with high‐throughput sequencing in a genome‐wide fashion, making it possible to probe the 3D architecture of genomes. Apart from the Hi‐C maps, the epigenetic annotations of certain locations such as compartments and TAD boundaries change significantly with the different environments and cell types.^[^ ^4^, ^5^, ^10^, ^11^, ^12^, ^13^ ^]^ For example, recent experimental and theoretical studies on the cell‐cycle process show that TADs and compartments vanish during mitosis.^[^ ^7^, ^14^, ^15^ ^]^ Therefore, the relationship between the ensemble behaviors and the epigenetics characteristics is important and in great needs for the investigations.

The pluripotent undifferentiated cell can differentiate into somatic cell. On the contrary, the mature somatic cell can be transformed into the induced pluripotent stem (iPS) cell with transcription factors by reprogramming.^[^ ^16^, ^17^ ^]^ Both differentiation and reprogramming can determine the cell fate by regulating the genome organizations and epigenetic characteristics. Moreover, one mature somatic cell can be transformed into another mature somatic cell directly without going through the iPS/stem cell state via the transdifferentiation process (or lineage reprogramming).^[^ ^18^, ^19^, ^20^ ^]^ This kind of technique can retain the signatures of the donor somatic cells with less risk of cancer.^[^ ^21^ ^]^ There are two major neural transdifferentiation approaches, the fibroblast cells (such as skin fibroblasts or peripheral blood mononuclear cells (PBMCs)) are transformed into neurons directly, or the fibroblast cells are converted into the induced neural progenitor cells (iNPCs) first and then to the neurons.^[^ ^22^, ^23^, ^24^ ^]^ Here in this study, we focus on the direct transdifferentiation processes between PBMC and bipolar neuron (BN), as well as the transdifferentiation processes between PBMC and neural progenitor cell (NPC), by using the method of chromosome modeling and molecular dynamic simulations. We set the starting and ending states as PBMC and BN. As a result, one pathway is the direct transition from PBMC to BN, while the other pathway is the transition from PBMC to BN through the intermediate NPC.

In recent years, Hi‐C experiments have made it possible to quantify the chromosome pairwise contact probability and 3D organization in vivo. However, the Hi‐C experiments have many limitations that they are costly to perform and the quality and reproducibility of Hi‐C data are determined by the sampling cells.^[^ ^25^, ^26^ ^]^ 3D chromosome models have been developed for simulations,^[^ ^27^, ^28^, ^29^, ^30^, ^31^, ^32^, ^33^, ^34^, ^35^, ^36^, ^37^, ^38^, ^39^ ^]^ most are the polymer models (homopolymer and heteropolymer models). The polymer models are often integrated with the data from Hi‐C, fluorescence in situ hybridization (FISH), or epigenetic marks in order to capture the 3D chromosome structures with hierarchies, territories, and genome functions. The Minimal Chromatin Model (MiChroM)^[^ ^36^ ^]^ is one of the heteropolymer models with different sub‐compartment types of polymer beads developed by Di Pierro et al. The parameters of this model were trained with the Hi‐C data of GM12878.^[^ ^36^ ^]^ Then, the MEGABASE^[^ ^37^ ^]^ was developed for establishing the relationship between the sub‐compartment annotations and the histone methylation and acetylation marks. This type of chromosome model by combining MiChroM and MEGABASE has been shown to successfully predict the 3D ensemble of chromosomal structures of different cell types that are consistent with the experimental Hi‐C data.^[^ ^37^, ^40^, ^41^, ^42^ ^]^ In addition, they presented a clear sequence‐to‐structure relationship between the sequences of epigenetic marks and genome architecture. Although molecular simulation provides microscopic structural description of individual molecule and gene expression dynamic provides mesoscopic description of gene dynamics, the link between the two is missing. Uncovering this link not only provides the microscopic description of gene expression and cell function, but also give rise to a solid physical foundation for the ab initio approach. Hi‐C experiments are performed using millions of cells at once, and report only a list of population averaged pairwise contact frequency *P*_*ij*_. The fluctuating characteristics of chromosome ensemble as well as the changing with epigenetics and gene expressions are the focus of this study.

Here in this study, we develop the chromosome structural ensemble switching model (CSESM) among several cell types, including the PBMC, BN, and NPC, to explore the pathways and the chromosome ensemble behaviors for the transdifferentiation processes. Consequently, we aim to investigate the underlying mechanisms of the transdifferentiation process, in particular the link between the ensemble behaviors and the epigenetic marks. We further show that the gene expression or the transcription activity is strongly correlated with the fluctuation of the local regions of the chromosome in ensemble. This uncovers the relationship among chromosome structures, ensemble fluctuations, epigenetics, and gene expressions. The ensemble behaviors can be quantified directly from the simulation data but not the average results from the Hi‐C data. Our simulation results provide a quantitative and intuitive measure of the Waddington's epigenetic landscape and uncover the fundamental relationship between chromosome ensembles and functions.

## 2 Results and Discussions

### 2.1 Two Phases of Chromatin

The previous microscopy assays have revealed that transcriptionally active euchromatic loci (compartment A) prefer to localize in the interior of nucleus, while the heterochromatin (compartment B) is mainly located at the nuclear periphery and the region surrounding the nucleoli.^[^ ^4^, ^43^, ^44^ ^]^ Importantly, recent observations strongly suggest that phase separation (microphase separation with the chromatin be regarded as a copolymer) is probably the essential mechanism of forming the two different kinds of chromatins/compartments.^[^ ^45^, ^46^, ^47^ ^]^ However, it is reported that the compartment A and compartment B have different compartmentalization mechanisms. The compartment B forms the heterochromatin phases mainly through HP1 protein and Polycomb complex.^[^ ^47^, ^48^ ^]^ And the compartment A forms the euchromatin phases mediated by the disordered regions of the active transcriptional factors (such as BRD4, TF15, and FUS), RNAs, RNA‐binding proteins, and RNA polymerases,^[^ ^49^, ^50^ ^]^ which are the necessary elements for transcription.

**Figure 1:**
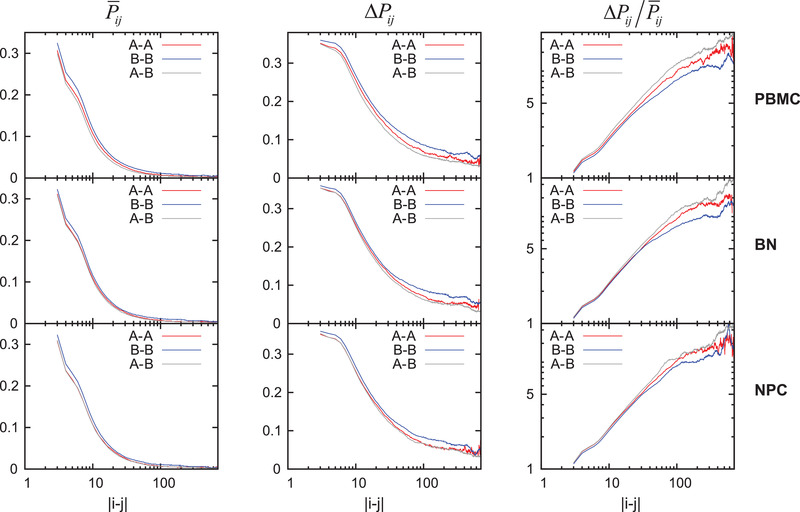
The mean contact probability $\bar{P_{ij}}$, the standard deviation Δ*P*_*ij*_, and the ratio of them $\Delta P_{ij}/\bar{P_{ij}}$ within the compartment A (A–A), within the compartment B (B–B), and between compartment A and B (A–B) as a function of the chain distance |*i* − *j*|. The *P*_*ij*_ is calculated with the distance between *i* and *j* (*r*_*ij*_): $P_{ij}=\frac{1}{2}\left(1+\tanh\left[\mu \left(r_{c}-r_{ij}\right)\right]\right)$. Here the mean and the standard deviation values are calculated among the chromosome ensemble.

In this study, we constructed an ensemble for the chromosome of different cells (PBMC, BN, and NPC), by using the 2800 frames extracted from the simulations. In order to compare the interactions within the compartment A/B and between the compartment A and the compartment B, we calculated the mean and the standard deviation of the contact probability *P*_*ij*_ ($\bar{P_{ij}}$ and Δ*P*_*ij*_) of the different interaction types (A–A, B–B, and A–B) in the ensemble. The characteristics of the interactions in different types indicate that the compartment A and the compartment B have different behaviors, corresponding to the two different phases of the chromosome (euchromatin and heterochromatin). The results of the $\bar{P_{ij}}$ in **Figure** **1** show that the compartment B has higher chromosome density than the compartment A (*P*_*ij*_ reflects the distance *r*_*ij*_ between *i* and *j*), consistent with the previous studies.^[^ ^49^, ^51^, ^52^ ^]^ This indicates more dense compaction for the compartment B and more sparse spread for the compartment A. It is obvious that the compartment B has higher Δ*P*_*ij*_ than the compartment A (see the middle panel of Figure 1). Considering that the contact probability *P*_*ij*_ is directly determined by the distance *r*_*ij*_ (see Section 4), a dimensionless quantity $\Delta P_{ij}/\bar{P_{ij}}$ is used to measure the relative fluctuation of the interactions in different compartments (the right panel of Figure 1 and Figure S1, Supporting Information). The results suggest that the compartment B is relatively more stable (lower $\Delta P_{ij}/\bar{P_{ij}}$) than the compartment A in the ensemble, with both higher $\bar{P_{ij}}$ and Δ*P*_*ij*_ values.

Moreover, we introduce a quantity named as “local chromosome fluctuation index (local CFI)” to measure the degree of the fluctuations of the locus *i* of chromosome in the ensemble: ${\mathrm{CFI}}_{i}=\frac{1}{N}\sum_{j}^{N}\Delta P_{ij}/\bar{P_{ij}}$, which is based on the fully sampled contact matrix. High local CFI corresponds to the relatively fluctuating region and low local CFI corresponds to the relatively stable region (in 50 kb resolution). The definition of local CFI is similar as the noise‐to‐signal ratio in the previous studies.^[^ ^25^ ^]^ As shown in Figure S2, Supporting Information, in simulation the local CFI is correlated with the two alternatives CFI^*α*^ and CFI^*β*^ (${\mathrm{CFI}}_{i}^{\alpha }=\frac{1}{N}\sum_{j}^{N}\sqrt{\left(1-\bar{P_{ij}}\right)/\bar{P_{ij}}}$; ${\mathrm{CFI}}_{i}^{\beta }=\frac{1}{N}\sum_{j}^{N}\sqrt{1/\bar{P_{ij}}}$), which are derived from the expressions and variants of noise‐to‐signal ratio. Here in this study, we take the H1‐hESC for an example to make comparison. There are 191829 contact pairs ($\bar{P_{ij}}$) from the Hi‐C data of chr21 for H1‐hESC (*q* arm, in 50 kb resolution), which are quite consistent with those from our simulations (squared Pearson correlation coefficient *R* ^2^ = 0.91). However, these contact pairs from Hi‐C are not continuous. A part of (*i*, *j*) pairs do not have values. The contact map calculated from simulation is continuous (220116 pairs in total). Every (*i*, *j*) pair has a *P*_*ij*_ value. We believe that the missing pairs in Hi‐C will affect the values of CFI^*α*^ and CFI^*β*^. For this reason, we make comparisons between the local CFI^*α*^ of H1‐hESC from simulation (continuous *P*_*ij*_) and the local CFI^*α*^ of H1‐hESC for selected pairs (discontinuous *P*_*ij*_). As shown in Figure S3, Supporting Information, the two kinds of CFI^*α*^ data have low correlations (*R* ^2^ = 0.35), even though they are both calculated from the simulations. Therefore, the continuous contact map is important for calculating the local CFI (or two alternatives CFI^*α*^ and CFI^*β*^). Consequently, the CFI^*α*^ or CFI^*β*^ from Hi‐C experiment does not correspond to that from simulation because of the inconsistent total pairwise contact number. In addition, not all of the cell‐types in this study have Hi‐C datasets. Therefore, we can not obtain the local CFI simply from the Hi‐C datasets.

**Figure 2:**
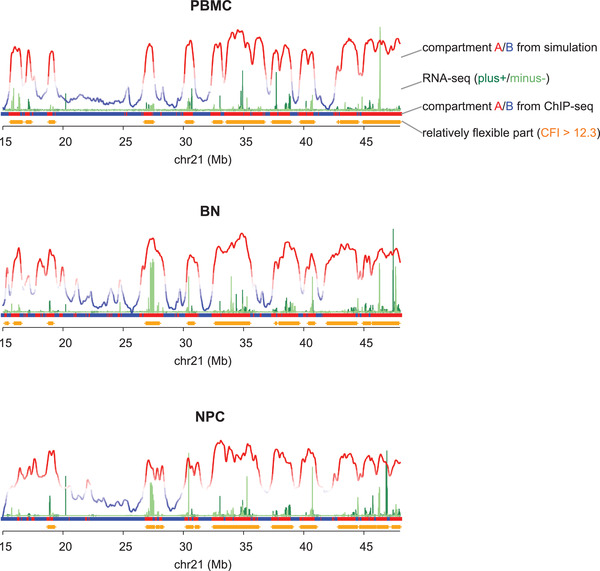
The compartment A/B (from simulation and from ChIP‐seq data), the RNA‐seq signals (from RNA‐seq data in ENCODE), and the relatively fluctuating part (quantified from simulation) of the chr21 (15–48 Mb) of PBMC, BN, and NPC, respectively; The compartment A/B (red/blue) from simulation was determined through the first principal component (pc1) of PCA. The part with relatively high local CFI (colored in orange) corresponds to the relatively fluctuating part in the ensemble.

In this study we have shown that the local CFI has a strong correlation with the gene expression (see Section 2.4). As a result, the local region with low CFI is relatively stable in the chromosome structural ensemble, corresponding to the compartment B, which is the gene‐silent part in nucleus. On the contrary, the local region with high CFI (CFI_i_ > 12.3 for chr21 and CFI_i_ > 13 for chr22) is relatively fluctuating in the chromosome structural ensemble, corresponding to the compartment A, which is the gene‐active part in nucleus. We checked and confirmed the results for both chr21 and chr22 of the different cells (shown in **Figure** **2** and Figure S4, Supporting Information). In addition, we have analyzed the mean distribution of each locus/monomer (*i*) along the chromosome radius in the ensemble (*ρ*_*max*_), which has the maximum radial distribution probability, as well as the ensemble‐averaged mean square displacement (MSD) of each monomer (*i*). As shown in Figure S5, Supporting Information, the local CFI is significantly correlated with the local monomer distribution (*ρ*_*max*_) and local monomer diffusivity (MSD). Compartment A (higher local CFI) tend to be localized at the outer layer of the chromosome and has higher diffusive ability than compartment B (lower local CFI), which is consistent with the previous results of chromosomes.^[^ ^36^, ^38^ ^]^ This provides a bridge between the microscopic chromosome structural stability/fluctuation and the transcription/gene expression activity.

### 2.2 Ensemble Fluctuation Reveals Transcription Activity and Stemness

**Figure 3:**
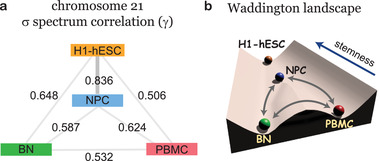
The squared Pearson correlation coefficient *γ* (*γ* = *R* ^2^) between the local CFI spectrums of two cells (a) and the schematic diagram of the Waddington landscape (b). In panel b, the balls represent the different cell types. And the possible transdifferentiation paths are labeled in gray arrows.

According to the Waddington's epigenetic landscape,^[^ ^53^, ^54^, ^55^ ^]^ the differentiated cells (here referred to the PBMC and BN) are located in the valleys of the Waddington's landscape, while the stem cells (here referred to H1‐hESC) are located at the top of the landscape hill. This implies that the H1‐hESC has much higher stemness than the other differentiated cells such as PBMC and BN. In addition, there are significant differences (low similarity) between the differentiated cells because they are located in the different valleys of the Waddington's landscape. In this study, we use the CFI spectrum (CFI_i_ values of the chromosome sequence) to make comparisons between different cells and show that the CFI spectrum can act as a key to quantify the similarity of sequence fluctuation distribution between the different cells. We calculated the squared Pearson correlation coefficient *γ* (*γ* = *R* ^2^) of the CFI spectrums to quantify the similarity between the two cells. As shown in **Figure** **3**a, the somatic cells PBMC and BN have low similarity (*γ* between PBMC and BN is 0.532), which shows that the two states are far different from each other. In contrast, the NPC has certain stemness as the similarity between it and H1‐hESC is high (*γ* between NPC and H1‐hESC is 0.836), much higher than the *γ* values between the somatic cells and H1‐hESC. We find that the results are consistent with the Waddington theory and we can obtain a similar landscape picture in terms of the locations of the stem cell, NPC, and differentiated cells (see Figure 3b), that NPC is located near the stem cell while the differentiated cells BN and PBMC are further away from the stem cell than NPC. Since the CFI spectrum represents the stability/fluctuation arrangement of the chromosome structural ensemble, we see that NPC shares more similar structural stability/fluctuation arrangement with the stem cell. We have illustrated that the local CFI has strong correlation with the compartment/epigenetic quantity (and gene expression, see Section 2.4). The results suggest that NPC and H1‐hESC have similar distribution of compartment A/B (and distribution of gene expression). Likewise, NPC/H1‐hESC has low similarity of the CFI spectrum with PBMC and BN, which means that the distribution of compartment A/B (and the distribution of gene expression) in NPC/H1‐hESC will be different from that in PBMC and BN. On the other hand, NPC shares more similar structural stability/fluctuation arrangement with differentiated cells than that between the differentiated cells. Our results suggest the important relationship among 1D, 3D, and function of the chromosome, which agrees with the conclusions in the previous studies.^[^ ^37^ ^]^

### 2.3 Transdifferentiation With Different Pathways

As the mentioned above, there are two different transdifferentiation approaches between PBMC and BN. One is the direct transdifferentiation from PBMC to BN; the other is the transdifferentiation from PBMC to BN via the intermediate state NPC (see Figure 3b). Aiming to analyze the detailed transdifferentiation path, we projected the system ensemble to different reaction coordinates. For compartment, we calculated the differences of pc1 (the first principle component of the normalized contact matrix) with respect to PBMC and BN and summed up all the difference absolute values (denoted as *δ*_PBMC and *δ*_BN), in order to quantify the epigenetic similarity between cells. Likewise, the correlation *γ* values with PBMC and BN (denoted as *γ*_PBMC and *γ*_BN) were used to quantify the ensemble similarity between cells. Since the compartment profiles are well correlated with the epigenetic profiles for the head and end points (NPC, BN, and PBMC cells) of each kinetic process, here the compartment profiles are considered to be correlated with the epigenetic profiles at every stage of the kinetic process.

**Figure 4:**
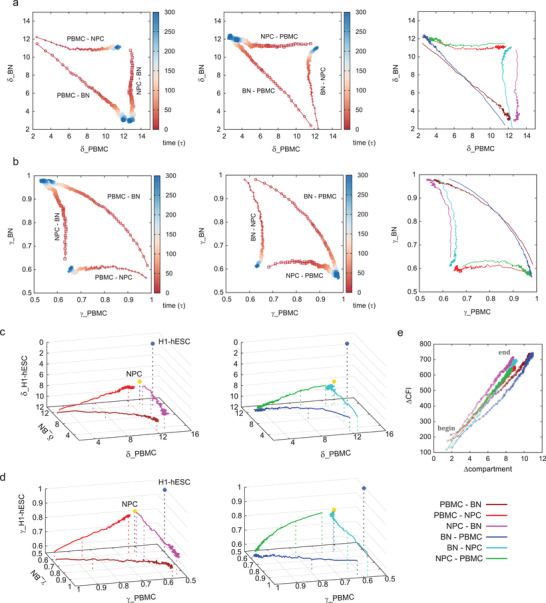
The kinetic pathways of the transdifferentiation processes between PBMC and BN, including the forward processes (left panels) PBMC–BN, PBMC–NPC, and NPC–BN, as well as the reverse processes (middle panels) BN–PBMC, BN–NPC, and NPC–PBMC. The trajectories were projected to two reaction coordinates: compartment differences (pc1) with respect to PBMC and BN (*δ*_PBMC and *δ*_BN, (a)), and the correlations with PBMC and BN (*γ*_PBMC and *γ*_BN, (b)); three reaction coordinates: *δ*_PBMC, *δ*_BN, and *δ*_H1‐hESC (c), *γ*_PBMC, *γ*_BN, and *γ*_H1‐hESC (d), respectively. The synchronization is shown via the variations of compartment and CFI (Δcompartment and ΔCFI, (e)). In panels a and b, the forward and reverse processes are combined in the right sides. The panels c and d reveal the Waddington epigenetic hill, with the stem cell H1‐hESC placed on the top of the hill. The locations of NPC, H1‐hESC, and the systems at time 1*τ* (start), 10*τ*, 300*τ* (end) are labeled. In panel e, Δcompartment and ΔCFI are the sum of the absolute differences of compartment (pc1) and CFI (local CFI spectrum) between time *τ* and time 0.

As shown in **Figure** **4**, kinetic simulation time 300 *τ* is long enough for reaching the terminal cell type BN because the *δ*_BN changes from about 12.0 to 3.0 and *γ*_BN changes from about 0.6 to 1.0 for both direct and indirect transdifferentiation. The paths of direct and indirect transdifferentiation are obviously different. The indirect transdifferentiation undergoes an intermediate state NPC with low similarity with both PBMC and BN, while the direct transdifferentiation does not pass through this location. In addition, from the results of time evolution of the transdifferentiation processes, the changes of the compartment and the local CFI are basically synchronized (shown in Figure 4e, Figures S6, S7, and S8, Supporting Information). We then compare the dynamical compartment and CFI from simulation with the RNA‐seq of PBMC and BN from experiment as there is a relationship among them (see Section 2.4). At time *τ* of each kinetic pathway, we obtained the distribution between gene expression (calculated as ln(RNA‐seq signal)) and compartment/CFI, and calculated the squared Pearson correlation coefficient (*R* ^2^) of the fitting line (*γ*_compartment‐gene and *γ*_CFI‐gene). As shown in Figure S9, Supporting Information, the changes of both compartment and CFI with respect of the gene expression are dynamically coupled.

The degree of the variations on both compartment and CFI of the direct transdifferentiation is higher than that of the indirect transdifferentiation (see Figure 4e). The significantly varying regions of the direct transdifferentiation (PBMC–BN and BN–PBMC) are wider distributed than that of the indirect transdifferentiation (PBMC–NPC, NPC–PBMC, NPC–BN, BN–NPC). For example, the regions after 38 Mb of the indirect transdifferentiation PBMC–NPC and NPC–PBMC, and the regions 27–32 Mb of the indirect transdifferentiation NPC–BN and BN–NPC do not have significant changes during the paths. It seems that the regions after 38 Mb are specific for BN‐related variations, and the regions 27–32 Mb are specific for PBMC‐related variations. We also calculated the paths of the reverse transdifferentiation processes from BN to PBMC. The forward and the reverse transdifferentiation processes have similar paths (see the right panels of Figure 4a,b).

Furthermore, aiming to characterize the Waddington's epigenetic landscape, a third reaction coordinate that quantifies the similarity to stem cell (H1‐hESC) was added to illustrate the 3D epigenetic map. As shown in Figure 4c,d, the somatic cells PBMC and BN are located in different valleys (high *δ*_H1‐hESC values and low *γ*_H1‐hESC values) compared with the stem cell (on top of the hill, *δ*_H1‐hESC=0 and *γ*_H1‐hESC=1). The NPC stays at the hill with medium *δ*_H1‐hESC and *γ*_H1‐hESC values (*δ*_H1‐hESC∼7.5 and *γ*_H1‐hESC∼0.8). This suggests that the indirect transdifferentiation can obtain certain stemness at the intermediate state, indicating an “uphill” path following with a “downhill” path on the Waddington's epigenetic landscape. However, the direct transdifferentiation processes are near the mountain valleys without significantly climbing on the epigenetic hill (with *δ*_H1‐hESC higher than 9.0 and *γ*_H1‐hESC lower than 0.7).

### 2.4 The Relationship among Epigenetics, Gene Expression, and Chromosome Ensemble Structure Fluctuations

**Figure 5:**
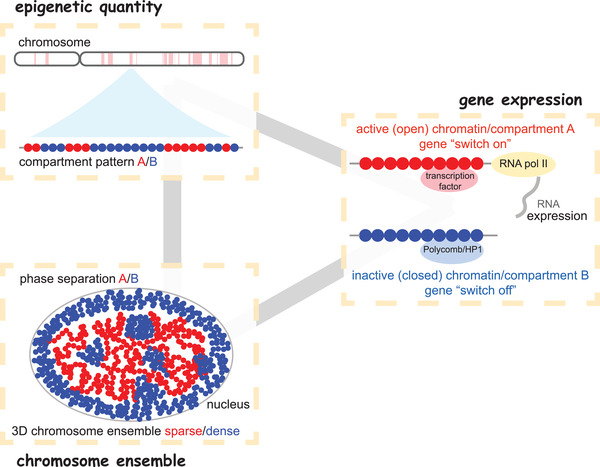
The scheme of the relationship among epigenetic quantity, gene expression, and chromosome ensemble, from the chromosome chain (1D) to the chromosome ensemble (3D). The chromosome chain can be seen as a copolymer with A (red) and B (blue) blocks. It can fold as a 3D structure in the interior of nucleus. The compartments A and B behave as different phases with different functions, that the compartment A (open chromatin) contains the transcription factory for the gene expressions while the compartment B is silent for the gene expressions.

**Figure 6:**
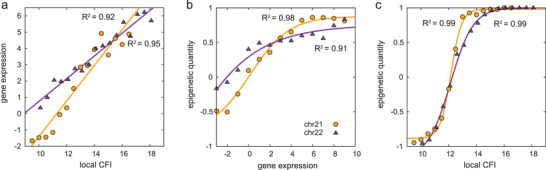
The correlation among epigenetic quantity, gene expression, and chromosome ensemble. a) The distribution of mean gene expression (calculated as ln(RNA‐seq signal)) versus the local CFI and the fit function, here the gene expression is quantified by firstly obtaining the natural logarithm of the RNA‐seq signals (ln(RNA‐seq signal)) of the loci with similar local CFI values then getting the mean value of these ln(RNA‐seq signal) values; b) the distribution of mean epigenetic quantity versus the gene expression and the fit function, the mean epigenetic quantity of the loci with similar gene expression is illustrated; c) the distribution of mean epigenetic quantity vs the local CFI and the fit function, the mean epigenetic quantity of the loci with similar local CFI is shown. The fit functions of panel a are *y* = 1.092*x* − 12.329 (chr21) and *y* = 0.697*x* − 6.222 (chr22). The fit functions of panel b are *y* = 1.672/(1 + *e* ^−0.550(*x* − 0.173)^) − 0.795 (chr21) and *y* = 6.446/(1 + *e* ^−0.265(*x* + 9.642)^) − 5.690 (chr22). The fit functions of panel c are *y* = 1.866/(1 + *e* ^−2.612(*x* − 12.165)^) − 0.885 (chr21) and *y* = 2.615/(1 + *e* ^−1.232(*x* − 12.138)^) − 1.162 (chr22).

Here in this study we uncover the crucial relationships among the chromosome ensemble (structural fluctuations), the epigenetic marks (ChIP‐seq), and the gene expressions (RNA‐seq), illustrated in **Figure** **5**. For one cell type without compartment being changed, our results support that the two compartments behave as different phases, with different fluctuations in the chromosome territories. However, we can not decide which compartment prefers to locate at the nuclear center or periphery because we only simulate one chromosome here. The relationship between the gene expression and the chromosome ensemble can be quantified via the simulation results that the ln(RNA‐seq signal) value increases linearly as the local CFI goes up (**Figure** **6**a). These results indicate that the phase of compartment A is the center of gene transcription/expression in the cell. The low density of this phase may be favorable for recruiting the essential transcription/expression partners, such as RNA, RNA‐binding proteins, and RNA pol II. The relatively fluctuation of the phase of the compartment A ensures the activity of gene transcription/expression (see Figures 1 and 2).

The sub‐compartment was defined at high resolution (25 kb) in situ Hi‐C experiment by Rao et al.^[^ ^5^ ^]^ The resolution of in situ Hi‐C contact map is as high as 1 kb. In this study, we compare the local CFI/gene expression results with experimental compartment A/B (Figure 6 and Figure S10, Supporting Information) by sorting sub‐compartments A1 and A2 (data from MEGABASE) as A, sub‐compartments B1, B2, and B3 as B, because we cannot determine the weights of these sub‐compartments when quantifying the compartment. The characteristics of different sub‐compartment patterns are shown in Figure S10, Supporting Information. There are obvious differences between compartment A and compartment B. While some sub‐compartment patterns, especially B2 and B3, do not have significant difference between them, which is consistent with the results of Rao et al. Therefore, we analyze the characteristics of chromosome A/B in this study. The compartment A/B can be quantified with +1 and −1 (epigenetic quantity) in this study (see Figure 5), which is similar to the positively charged and negatively charged residues of a polyampholyte chain.^[^ ^56^, ^57^, ^58^ ^]^ We found that there are much more loci with low local CFI values (or low gene expressions) have −1 epigenetic quantity than those with high local CFI values (or high gene expressions). Then in order to make clear comparison, we calculated the distribution of the epigenetic quantity on the gene expressions. It is obvious that there is a correlation (sigmoid type) between the gene expression and the mean epigenetic quantity (see Figure 6b). And Figure 6c clearly shows the two phases of compartment A and compartment B, corresponding to high and low local CFI values, respectively. The sigmoid type function (1/(1 + *e* ^−*μ*(*x* − *a*)^) type) represents the two phases of the compartments A and B better, which shows a sharp slope on the distribution data. Larger *μ* parameter corresponds to sharper slope between the platform regions. The epigenetic quantity is like a switch between −1 and +1. Therefore, we describe the relationship between epigenetic quantity and gene expression, between epigenetic quantity and local CFI as the sigmoid type function because the chromatin locus tends to be compartment A (gene “switch on”) when the gene expression/local CFI is high. In addition, we added the distribution data of one cell‐type each. As shown in Figure S11, Supporting Information, the results suggest that these correlations are not cell‐type‐dependent. Different chromosomes will have a slight effect on the fitting slope and intercept, but not the trend of the fitting line. Moreover, the sequence charge decoration (SCD) value can reveal the property of the monomer distribution ($\mathrm{SCD}=\frac{1}{N}\sum_{i=1}^{N}\sum_{j=i+1}^{N}\alpha_{i}\alpha_{j}\sqrt{j-i}$).^[^ ^56^, ^58^ ^]^ The value of *α* is assigned according to the compartment or local CFI. SCD with large absolute value implies the sequence with charge‐blocky pattern, SCD with small absolute value implies the sequence with charge‐scrambled pattern (see sv15 and sv1 sequences in ref. [56]). Here we calculated the SCD values for the epigenetic quantity spectrum and the local CFI spectrum for different sequences (see Section 4). The results suggest that a strong linear correlation between the distributions of epigenetic quantity and chromosome ensemble (Figure S12, Supporting Information). In addition, we have only four cell types in the current study, which is not abundant enough to make accurate correlation in one chromosome. Therefore, we added two other cell types, GM12878 (GM21) and IMR90 (IMR21) to calculate the correlation between the two kinds of SCD values of chr21 (see Figure S12, Supporting Information, cyan line). The results do suggest that a strong linear correlation between the distributions of epigenetic quantity and local CFI.

Consequently, the results and discussions above demonstrate that there is a close connection among the epigenetic quantity, the chromosome ensemble, and the gene expression (Figure 5). The distribution of epigenetic quantity can influence the chromosome fluctuation in the structure ensemble and the parts involved in the gene transcriptional factories. The chromosome behaviors in the ensemble determine the phase separation of the compartment A/B (epigenetics), as well as the ratio of active/silent genes. Meanwhile, the changes on gene expressions/proteins can influence the level of the histone modifications,^[^ ^59^ ^]^ therefore may have an effect on the chromosome behaviors in the ensemble.

For the transdifferentiation processes between PBMC and BN, we have explored the transdifferentiation mechanisms as well as the paths on the Waddington landscape. In experiments, the transdifferentiation is realized with the transcriptional factors.^[^ ^18^, ^19^, ^20^ ^]^ The active transcriptional factors can bind to a region of the heterochromatin with inactive genes, transforming it to the euchromatin. Or certain transcriptional factors can competitively bind to a region of the active euchromatin, switching it to the inactive heterochromatin (B–A or A–B transition). We notice that the changes of the epigenetics (such as the compartment switching A–B or B–A) are not necessarily accompanied with the changes on the genes (DNA sequences). This can have an impact on both the chromosome ensemble and the gene expression. As mentioned above, the changes on the distribution of the epigenetic quantity will have a significant impact on the chromosome behaviors in the ensemble as well as the parts involved in the gene transcriptional factories, eventually leading to a completely different cell fate.

## 3 Conclusion

In this study, we uncover the quantitative relationship among epigenetics, gene expression, and chromosome ensemble structural fluctuations by developing the chromosome structural ensemble switching model (CSESM) and performing the simulations on different cell types. The distribution of both epigenetic quantity and chromosome fluctuation have linear correlations with the gene expression. We show the phase separation of the compartments A and B with different behaviors and functions in the chromosome territories. The compartment A is the center of gene expression, with lower density and higher fluctuation than that of the compartment B. In addition, for the transidifferentiation processes between PBMC and BN, we have explored the transidifferentiation mechanisms as well as the paths on the Waddington landscape. The stem cell is located on the top of the Waddington landscape, the differentiated cells PBMC and BN are located in different valleys, while the NPC is located between the stem cell and the differentiated cell. The path of direct transidifferentiation is obviously different with that of the transidifferentiation via the intermediate state NPC, which shows an “uphill” process following with a “downhill” process. These theoretical studies provide microscopic molecular and structural insights on the chromosome fluctuations/ensembles, and the interplay with the epigenetics and gene expressions in determining the cell fate.

## 4 Experimental Section

### Chromosome Models and Simulation Settings

We used the models from Nucleome Data Bank (NDB, https://ndb.rice.edu)^[^ ^40^ ^]^ to build the initial structures and potential energies of chromosomes 21 and 22, for different types of human cells (H1‐hESC for stem cell, bipolar neuron (BN), neural progenitor cell (NPC), and peripheral blood mononuclear cell (PBMC)). The NDB enables physics‐based chromosome models for molecular dynamic simulations, by combining the MEGABASE and MiChroM computational pipelines.^[^ ^36^, ^37^ ^]^ The MiChroM model is a 3D chromatin polymer chain at 50 kb resolution, including the epigenetic information from the Encode Project database (epigenetic marking patterns of compartments and sub‐compartments from ChIP‐seq assays). Therefore, it is a type of block‐copolymer (heteropolymer) model with given potential form

(1) $$\begin{array}{ccc} U_{MiChroM}\left(\vec{r}\right) & = & U_{HP}\left(\vec{r}\right)+\sum_{\begin{array}{c} k\geq l\\ k,l\in \text{Types} \end{array}}\alpha_{kl}\sum_{\begin{array}{c} i\in \{\text{Loci}\;\text{of}\;\text{Type}\;k\}\\ j\in \{\text{Loci}\;\text{of}\;\text{Type}\;l\} \end{array}}f\left(r_{ij}\right)\\ & & +\chi \cdot \sum_{\left(i,j)\in \{\text{Loop}\;\text{Sites}\right\}}f\left(r_{ij}\right)+\sum_{d=3}^{500}\gamma \left(d\right)\sum_{i}f\left(r_{i,i+d}\right), \end{array}$$

where *U*_*HP*_ is the homo‐polymer potential term of the chromosome connectivity, in resolution of 50 Kb of DNA, the second term describes the type–type interactions of the chromosome where the type is the compartment annotation determined by MEGABASE, the third term is the interactions between loop anchors that are related to the protein CTCF, the final term is the one referred to the ideal chromosome model. Further details for this model can be referred to the reference of NDB.^[^ ^40^ ^]^ This model is transferable for different chromosomes and cells, and has been shown to successfully predict the Hi‐C contact maps of multiple human cell lines, and is consistent with the fluorescence in situ hybridization (FISH) experimental results.^[^ ^40^, ^41^, ^42^ ^]^ This chromosome model in 50 kb resolution is able to show the polymeric properties of chromosomes, as well as the compartment characteristics (intra‐ and inter‐chromosome). In addition, with different parameters on the sub‐compartments encoded in the model (heteropolymer), this model can quantify the detailed global architecture, the compartment, and the phase separation of the chromosomes. Higher resolution model (∼5 kb) is needed for characterizing the human genes (average size about 30 kb),^[^ ^60^ ^]^ chromatin loops, TADs (10 kb to 1 Mb), promoters, and enhancers.^[^ ^61^ ^]^

Here in this study, the 935‐ and 1017‐bead models (*n* = 935 and *n* = 1017), representing the human chromosomes 21 and 22 were prepared for molecular dynamics (MD) simulations, respectively. The simulations were performed by Gromacs 2018^[^ ^62^ ^]^ with reduced units. Langevin stochastic dynamics were applied with a friction coefficient of 1.0 *τ* ^−1^, where *τ* is the reduced time unit. In order to obtain the ensemble‐average properties of the chromosomes, such as the probability contact maps and the fluctuations, we constructed the ensemble of chromosome after a two‐stage simulation process:^[^ ^35^ ^]^ a heating stage (2 × 10^5^ *τ* at temperature 3.0 in reduced time unit) to relax the initial model inside the nucleus wall as well as a replica‐exchange stage (1 × 10^5^ *τ* of 28 replicas, all at about 1.0 temperature in reduced time unit) to enhance the sampling of the chromosome structures. Then an ensemble of 2800 chromosome frames were collected from the last 5 × 10^4^ *τ* of the 28 replicas (100 frames each).

We developed the chromosome structural ensemble switching model (CSESM), which is similar as the previous energy landscape‐switching model,^[^ ^15^, ^63^, ^64^ ^]^ to explore the dynamical processes between different kind of cell‐types. By encoding the epigenetic information in the chromosome model, we performed the transdifferentiation processes from PBMC to BN and from BN to PBMC. From a physical perspective, the energy landscapes of two cell types of the transdifferentiation will differ significantly, form structural to energy. The energy excitation–relaxation landscape‐switching model is developed for the non‐equilibrium process to connect the two distinct energy landscapes. The non‐equilibrium effects in the landscape‐switching model are the driving forces for the transdifferentiation processes, which is often in the form of extended ATP hydrolysis for energy pumping in biology. As the previous simulation procedures,^[^ ^15^, ^63^, ^64^ ^]^ here the CSESM simulations were performed under the potential of the beginning state of transdifferentiation. Then the potential was suddenly switched to that of the end state of transdifferentiation. Finally, simulations were performed under the potential of the end state of transdifferentiation. In this study, there are two different pathways for the transdifferentiation process: one is the direct one‐step transdifferentiation (PBMC–BN or BN–PBMC), and the other is the two‐step transdifferentiation via an intermediate state (PBMC–NPC–BN or BN–NPC–PBMC). In order to obtain the properties of the chromosome ensemble, 2800 runs were performed for each transdifferentiation step. For each transdifferentiation step, 1.5 × 10^3^ *τ* is long enough for one type of chromosome ensemble to transform to another. We can quantify the degree of the chromosome fluctuations and obtain the important information on the chromosome ensemble at each time step.

### Statistical Analysis

For each chromosome ensemble, the probability contact map *P* can be calculated as $P_{ij}=\frac{1}{2}\left(1+\tanh\left[\mu \left(r_{c}-r_{ij}\right)\right]\right)$, where *μ* = 3.22 and *r*_*c*_ = 1.78 were set according to the previous studies.^[^ ^35^, ^36^, ^37^, ^40^ ^]^ According to the probability contact map *P*, the A and B compartments were identified via the same method used by Lieberman‐Aiden et al.^[^ ^4^, ^65^, ^66^ ^]^ That is, the enhanced probability contact map, calculated by the observed/expected matrix, was normalized by ICE method and then converted into a Pearson correlation matrix. The compartment profiles were determined by the first principal component (pc1) of the principal component analysis (PCA) of the matrix. Within each ensemble, the relative fluctuation of a contact pair between *i* and *j* was measured via a dimensionless quantity $\Delta P_{ij}/\bar{P_{ij}}$, where the Δ*P*_*ij*_ and $\bar{P_{ij}}$ are the standard deviation and the mean of all the *P*_*ij*_ among the chromosome ensemble. Then we use the local chromosome fluctuation index (local CFI) to quantify the degree of the fluctuations of the chromosome at monomer *i* in the ensemble: ${\mathrm{CFI}}_{i}=\frac{1}{N}\sum_{j}^{N}\Delta P_{ij}/\bar{P_{ij}}$. The CFI spectrum (CFI_i_ values of the chromosome) quantifies the fluctuation distribution of the chromosome.

In order to analyze the distribution of each locus/monomer of the chromosome, we calculated the radial distribution function (rdf) with respect to the center of the chromosome and found the position with the highest probability. Then we can obtain the time‐averaged distribution of monomer radial position *ρ*_*max*_ (in reduced unit). The mean square displacement (MSD) can be calculated to characterize the dynamics/diffusions of the monomer *i* by the Einstein relation. The ensemble‐averaged MSD was obtained through the last half trajectory (5 × 10^4^ *τ*) of each replica.

For the 1D chromosome chain with A/B epigenetic marks, we assigned +1 (*α*_*i*_ = 1) for compartment A and −1 (*α*_*i*_ = −1) for compartment B. Then the sequence charge decoration (SCD) values were calculated for the chromosome chains of chr21 and chr22 ($\mathrm{SCD}=\frac{1}{N}\sum_{i=1}^{N}\sum_{j=i+1}^{N}\alpha_{i}\alpha_{j}\sqrt{j-i}$).^[^ ^56^, ^58^ ^]^ We also calculated the SCD values for the CFI spectrums of chr21 and chr22, with high local CFI part (CFI_i_ > 12.3 in chr21, CFI_i_ > 13 in chr22) set as +1 and low local CFI part set as −1. The threshold values correspond to about 60% level from the highest CFI_i_ to the lowest CFI_i_. Here SCD with large absolute value means the sequence with charge‐blocky pattern, SCD with small absolute value means the sequence with charge‐scrambled pattern.^[^ ^56^ ^]^

### RNA‐seq Data

All the RNA‐seq (total RNA‐seq) data were downloaded from ENCODE,^[^ ^2^ ^]^ including the BN (ENCSR968WKR), NPC (ENCSR244ISQ), and PBMC (ENCSR000CUT). Both plus and minus strands signal of all reads were used for comparison.

## Conflict of Interest

The authors declare no conflict of interest.

## Supporting information

Supporting Information

## Data Availability

The data that support the findings of this study are available from the corresponding author upon reasonable request.
