# Supplementary material for: Uncovering the Quantitative Relationships Among Chromosome Fluctuations, Epigenetics, and Gene Expressions of Transdifferentiation on Waddington Landscape
Source: Adv Sci (Weinh). 2022 Feb 1;9(10):2103617. doi: 10.1002/advs.202103617 (PMC8981899; doi:10.1002/advs.202103617)
Supplement: Supplementary file 1 — Supporting Information [file ADVS-9-2103617-s001.pdf]

ADVANCED SCIENCE
Open Access

Supporting Information

for *Adv. Sci.*, DOI 10.1002/advs.202103617

Uncovering the Quantitative Relationships Among Chromosome Fluctuations, Epigenetics, and Gene Expressions of Transdifferentiation on Waddington Landscape

*Wen-Ting Chu, Xiakun Chu and Jin Wang**

ADVANCED SCIENCE
Open Access

Supporting Information

for *Adv. Sci.*, DOI: 10.1002/advs.202103617

Uncovering the quantitative relationships among chromosome fluctuations, epigenetics, and gene expressions of transdifferentiation on Waddington landscape

*Wen-Ting Chu Xiakun Chu Jin Wang**

Supporting information:
Uncovering the quantitative relationships among chromosome fluctuations, epigenetics, and gene expressions of transdifferentiation on Waddington landscape

December 21, 2021

1 Figures

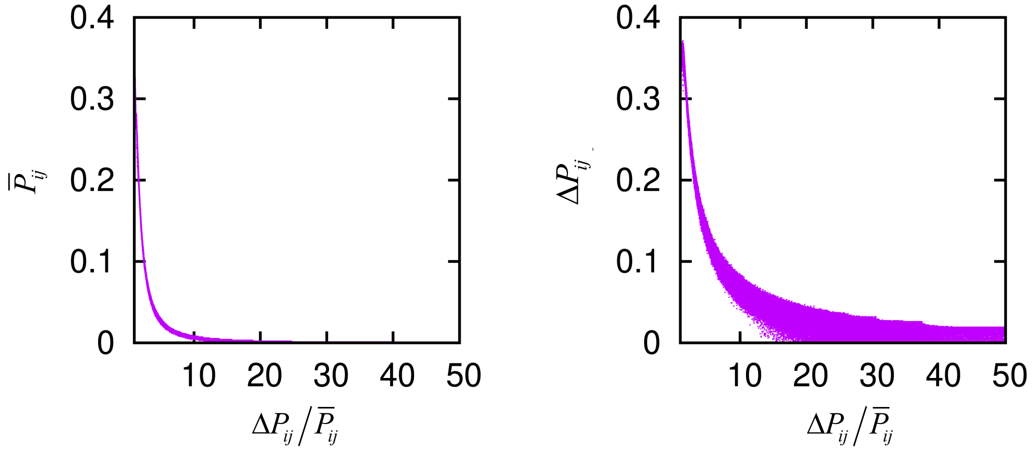

Fig. S1 $\overline{P_{ij}}$ vs $\Delta P_{ij}/\overline{P_{ij}}$ as well as ΔP_{ij} vs $\Delta P_{ij}/\overline{P_{ij}}$. Both $\overline{P_{ij}}$ and ΔP_{ij} decrease sharply as the $\Delta P_{ij}/\overline{P_{ij}}$ increases.

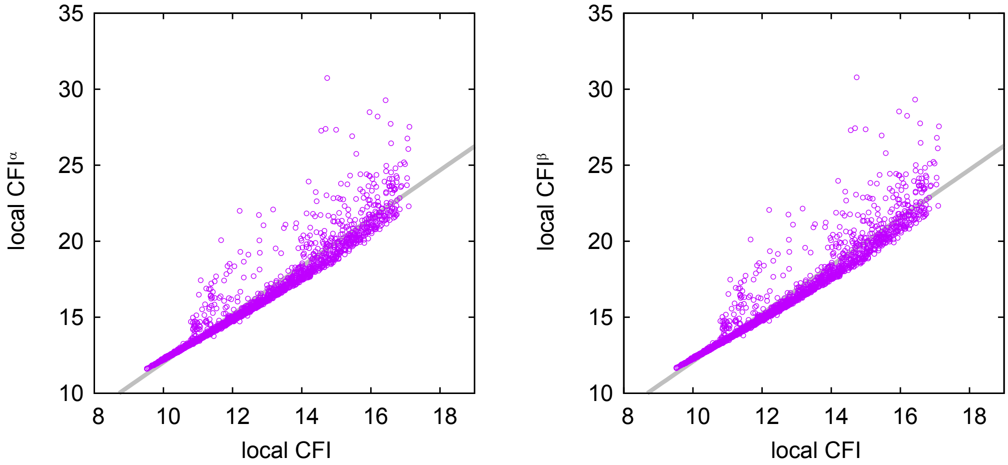

Fig. S2 Local CFI vs local CFI$^\alpha$ as well as local CFI vs local CFI$^\beta$ ($\mathrm{CFI}_\mathrm{i}^\alpha = \frac{1}{N}\sum_j^N \sqrt{\left(1-\overline{P_{ij}}\right)/\,\overline{P_{ij}}}$; $\mathrm{CFI}_\mathrm{i}^\beta = \frac{1}{N}\sum_j^N \sqrt{1/\,\overline{P_{ij}}}$). Both CFI$^\alpha$ and CFI$^\beta$ are from the expressions and variants of noise-to-signal ratio. All the linear fitting R^2 values are higher than 0.91.

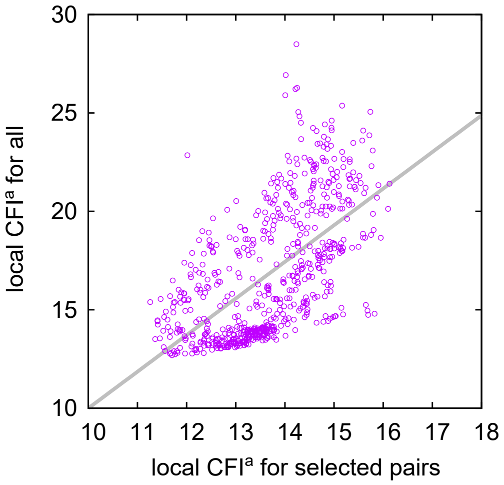

Fig. S3 The local CFI$^{\alpha}$ for selected pairs (191829 Hi-C (i, j) pairs) vs the local CFI$^{\alpha}$ for all pairs (220116 in total). Here all the CFI$^{\alpha}$ data are calculated from the P_{ij} in simulations.

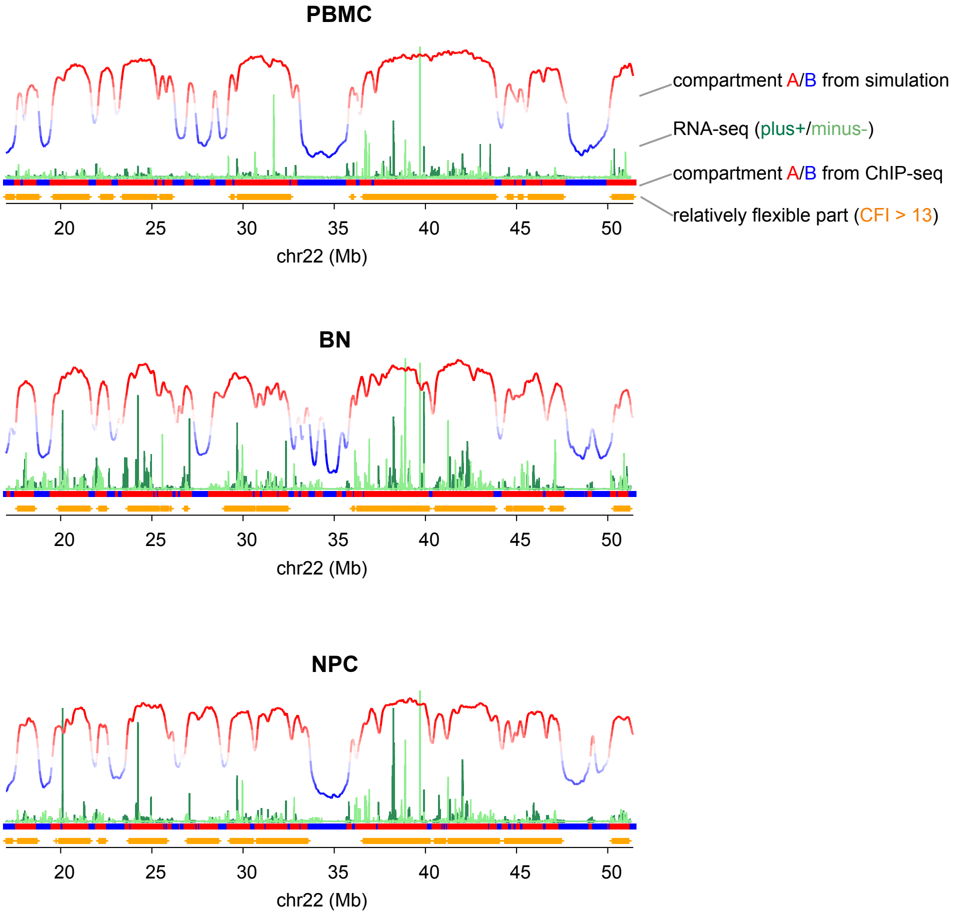

Fig. S4 The compartment A/B (from simulation and from ChIP-seq data), the RNA-seq signals (from RNA-seq data in ENCODE), and the relatively fluctuating part (quantified from simulation) of the chr22 (17–51.4 Mb) of PBMC, BN, and NPC, respectively; The compartment A/B (red/blue) from simulation was determined through the first principal component (pc1) of PCA. The part with relatively high local CFI (colored in orange) corresponds to the relatively fluctuating part in the ensemble.

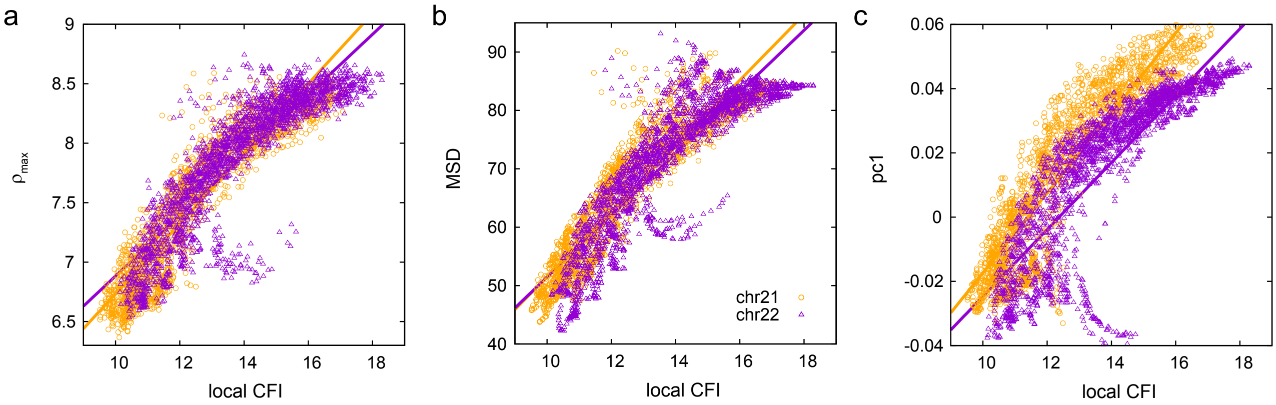

Fig. S5 The correlation between local CFI and local monomer distribution (ρ_{max}, *a*), between local CFI and local monomer diffusivity (MSD, *b*), between local CFI and epigenetics (compartment from simulation, pc1, *c*). The fit functions of panel *a* are $y = 0.294x + 3.791$ (chr21) and $y = 0.254x + 4.343$ (chr22). The fit functions of panel *b* are $y = 5.613x + 4.635$ (chr21) and $y = 5.284x + 1.384$ (chr22). The fit functions of panel *c* are $y = 0.0124x - 0.142$ (chr21) and $y = 0.0104x - 0.129$ (chr22). All the squared Pearson correlation coefficient R^2 values are higher than 0.7.

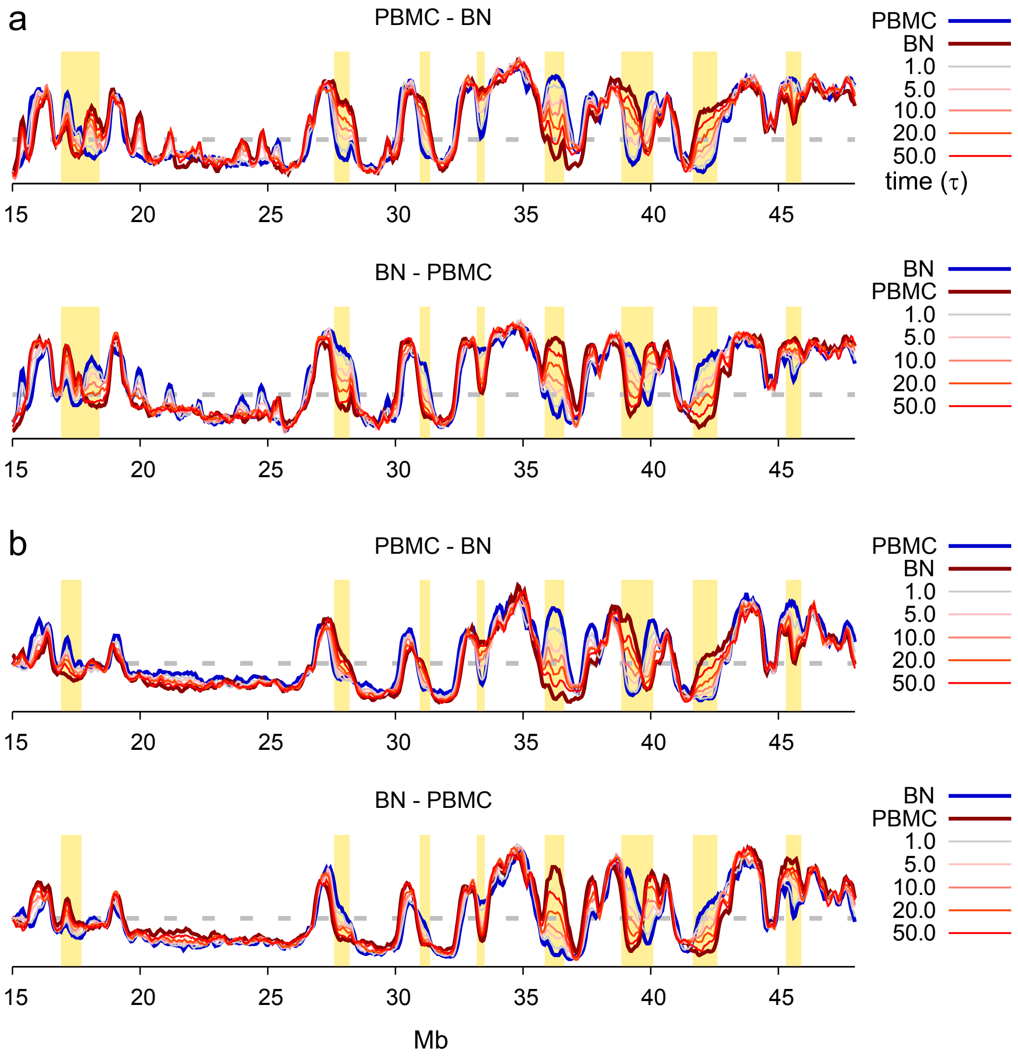

Fig. S6 The compartment (*a*) and the local CFI (*b*) spectrum of the different cell types in the transidifferentiation processes PBMC–BN and BN–PBMC, including the beginning and the end cell types, as well as the frames at 1.0, 5.0, 10.0, 20.0, and 50.0 τ. Values 0 (compartment, *a*) and 12.3 (local CFI, *b*) are used to characterize different types of chromatin. The regions with obvious changes during transidifferentiation are highlighted in yellow.

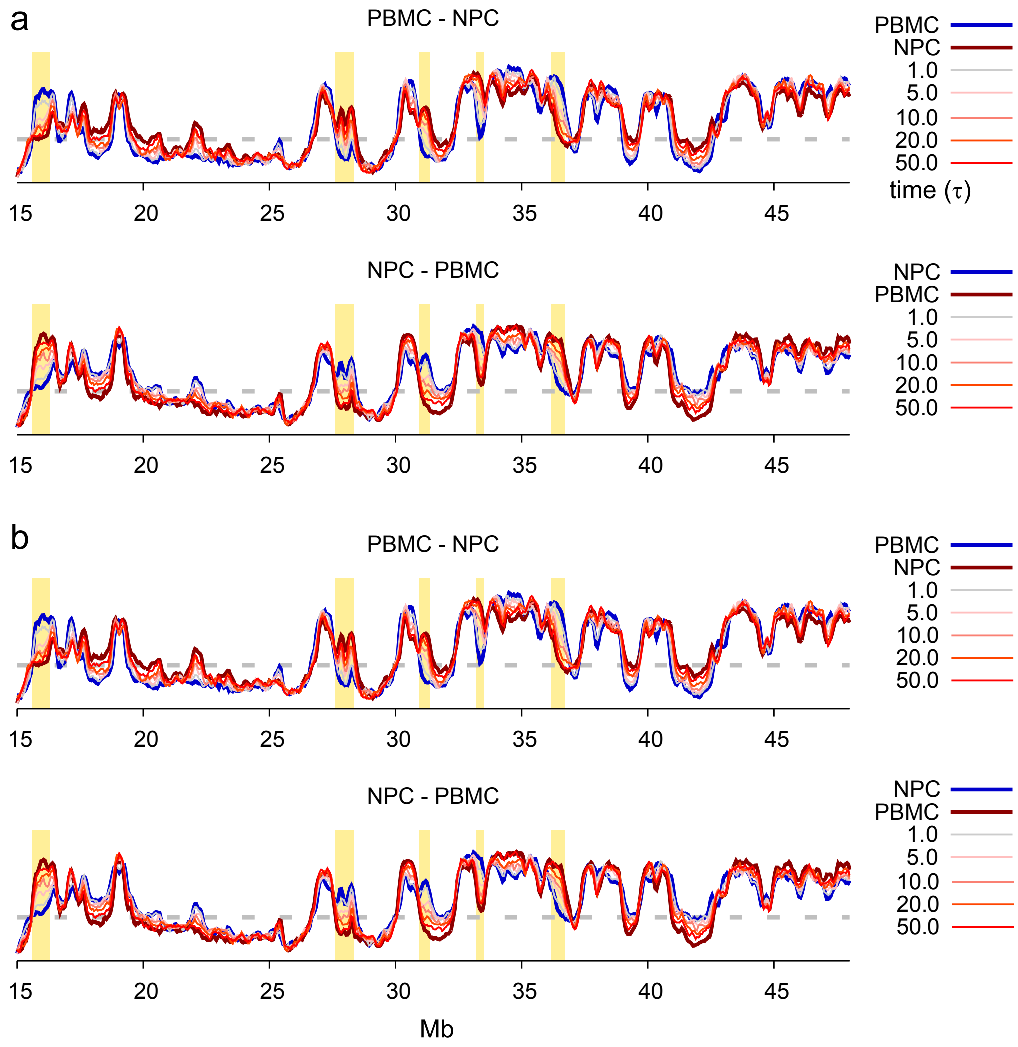

Fig. S7 The compartment (*a*) and the local CFI (*b*) spectrum of the different cell types in the transidifferentiation processes PBMC–NPC and NPC–PBMC, including the beginning and the end cell types, as well as the frames at 1.0, 5.0, 10.0, 20.0, and 50.0 τ. Values 0 (compartment, *a*) and 12.3 (local CFI, *b*) are used to characterize different types of ch romatin. The regions with obvious changes during transidifferentiation are highlighted in yellow.

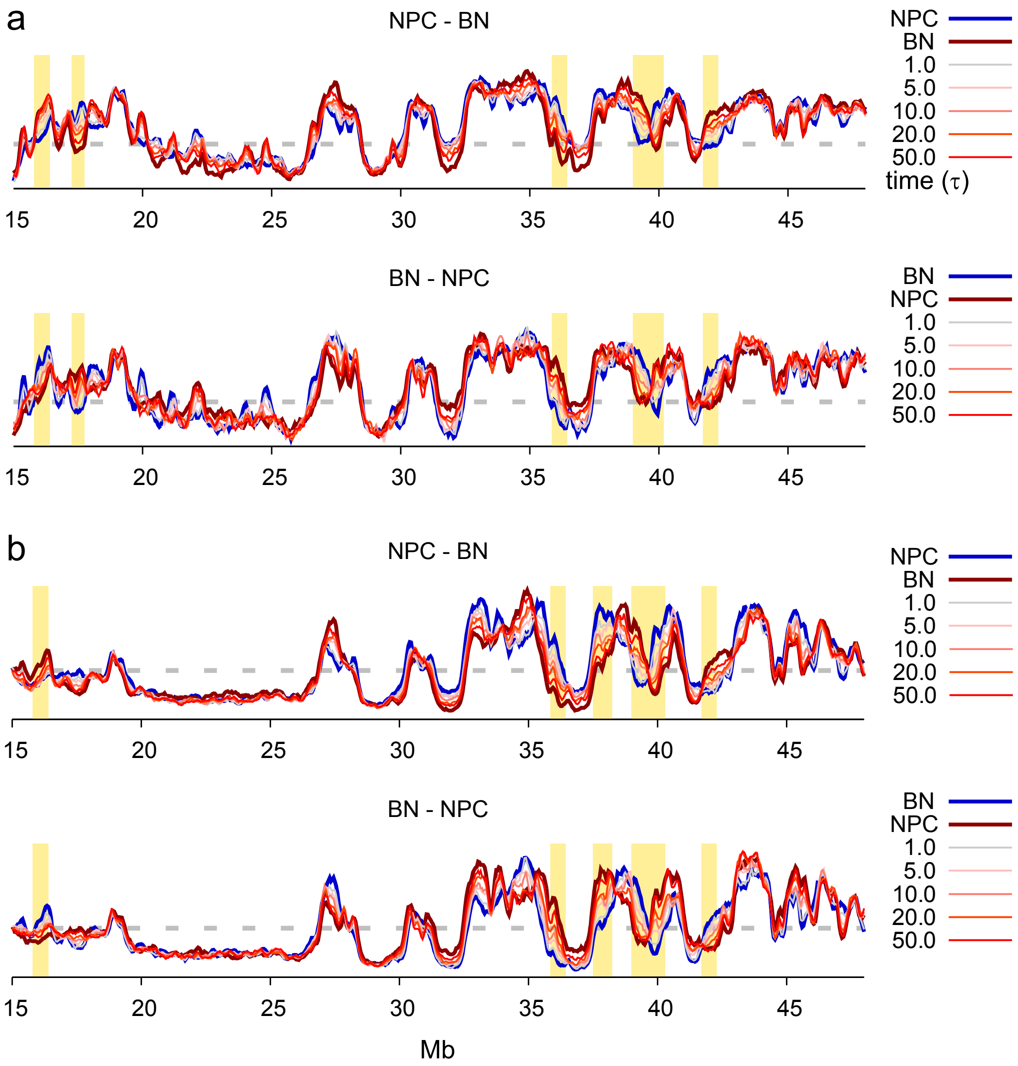

Fig. S8 The compartment (*a*) and the local CFI (*b*) spectrum of the different cell types in the transidifferentiation processes NPC–BN and BN–NPC, including the beginning and the end cell types, as well as the frames at 1.0, 5.0, 10.0, 20.0, and 50.0 τ. Values 0 (compartment, *a*) and 12.3 (local CFI, *b*) are used to characterize different types of ch romatin. The regions with obvious changes during transidifferentiation are highlighted in yellow.

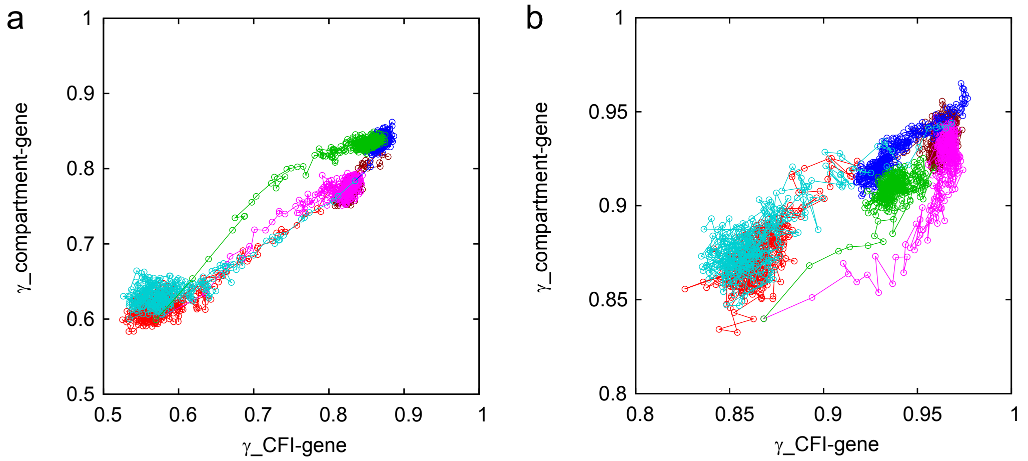

Fig. S9 The coupling between CFI and compartment with respect of gene expression of PBMC (*a*) and BN (*b*). γ_compartment-gene and γ_CFI-gene are the correlations between the distributions of compartment and RNA-seq, between the distributions of CFI and RNA-seq. Different transdifferentiation processes are colored the same as that in Figure 4.

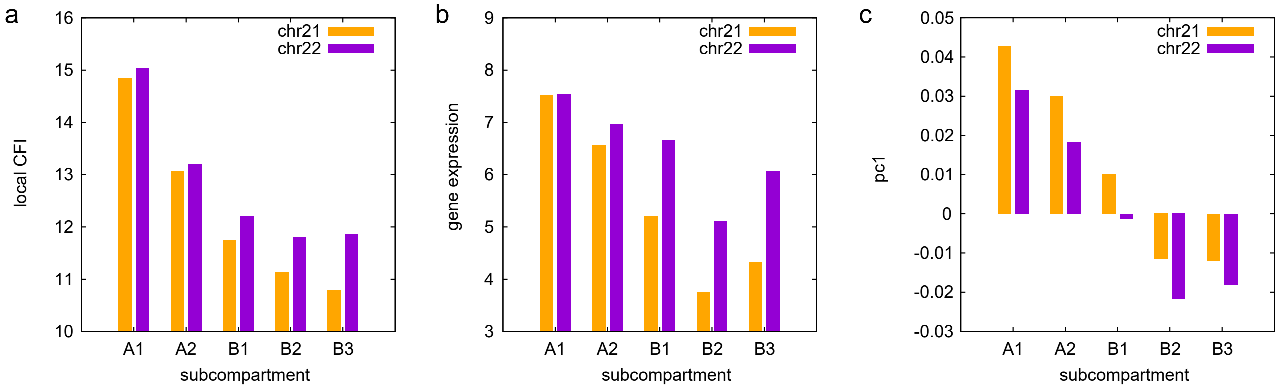

Fig. S10 The local CFI (*a*), gene expression (*b*), compartment from simulation (pc1, *c*) of different sub-compartment patterns. The sub-compartment patterns are from the MEGABASE (NDB, https://ndb.rice.edu).

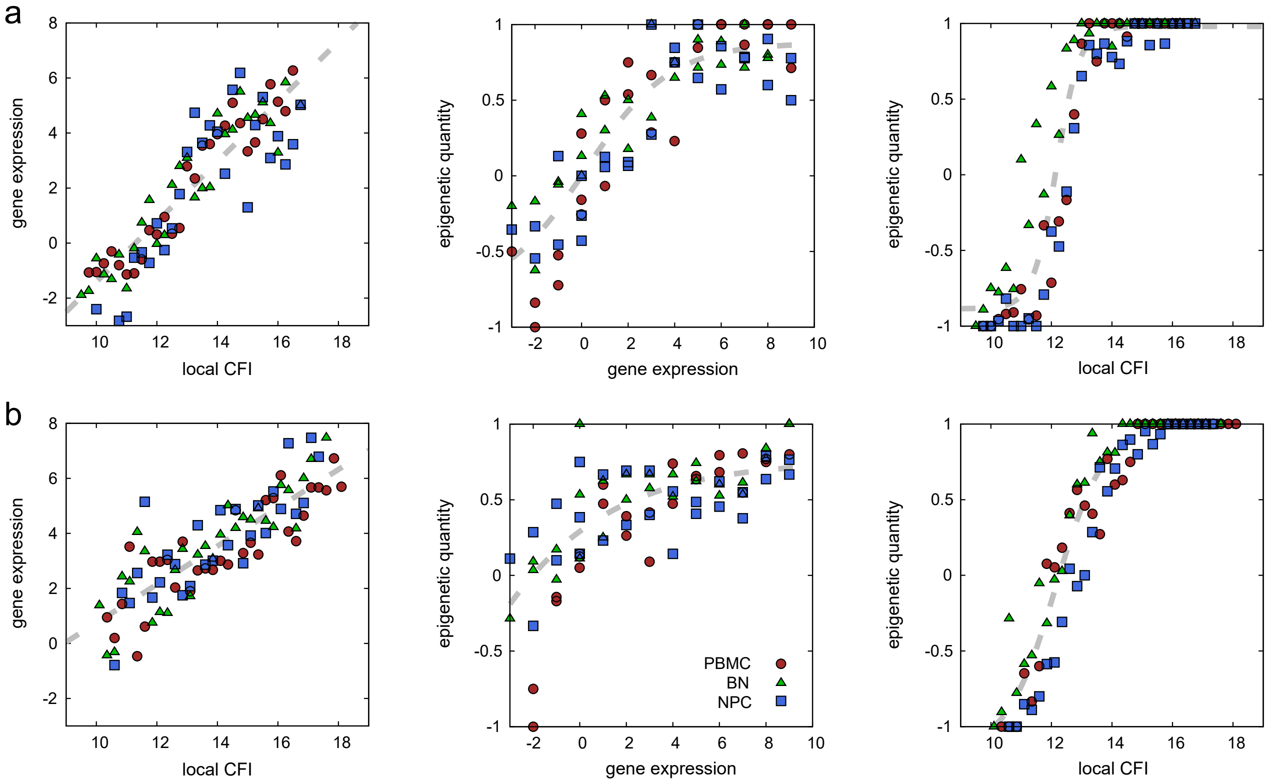

Fig. S11 The distribution data of epigenetic quantity, gene expression, and chromosome ensemble in different cell-types including PBMC, BN, and NPC (*a*, chr21; *b*, chr22). The grey dashed lines are the fitting lines in Figure 6.

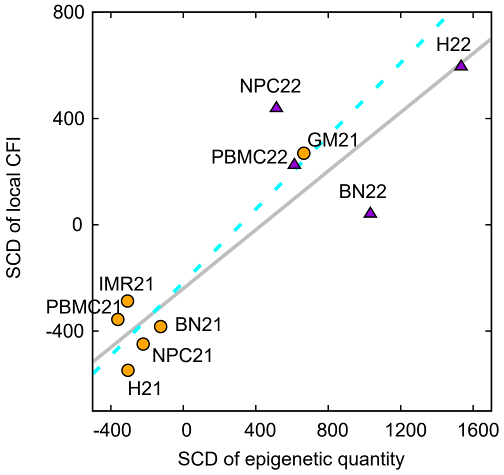

Fig. S12 The SCD of epigenetic quantity vs the SCD of local CFI and the fit function ($y = 0.553x - 240.022$, gray line).
